# Molecular and Histological Identification of Bovine Papillomavirus 1, 2 and a Novel Genotype in Cutaneous Papillomas of Dairy Cattle in Taiwan

**DOI:** 10.1155/tbed/5586786

**Published:** 2025-06-25

**Authors:** Guan-Shiun Chen, Hue-Ying Chiou, Yen-Chen Chang, Hao-Ping Liu, Yu-I Pan, Ming-Yun Chan, Tsung-Ching Liu, Ming-Yuan Chia, Chienjin Huang, Jacky Peng-Wen Chan, Chia-Yu Chang

**Affiliations:** ^1^Department of Veterinary Medicine, College of Veterinary Medicine, National Chung Hsing University, Taichung City 402, Taiwan; ^2^Graduate Institute of Veterinary Pathobiology, College of Veterinary Medicine, National Chung Hsing University, Taichung City 402, Taiwan; ^3^Graduate Institute of Molecular and Comparative Pathobiology, School of Veterinary Medicine, National Taiwan University, Taipei 106, Taiwan; ^4^Department of Veterinary Medicine, College of Veterinary Medicine, National Pingtung University of Science and Technology, Pingtung 912, Taiwan; ^5^Veterinary Medical Teaching Hospital, Department of Veterinary Medicine, College of Veterinary Medicine, National Pingtung University of Science and Technology, Pingtung 912, Taiwan; ^6^Department of Animal Science and Biotechnology, Tunghai University, Taichung 407, Taiwan; ^7^Graduate Institute of Microbiology and Public Health, College of Veterinary Medicine, National Chung Hsing University, Taichung City 402, Taiwan

**Keywords:** bovine papillomavirus (BPV), cattle, fibropapilloma, in situ hybridization (ISH), *Xipapillomavirus*

## Abstract

Bovine papillomaviruses (BPVs) are host-specific and strongly epitheliotropic infectious agents that cause benign epithelial and mucosal proliferations, with potential for malignant transformation. However, BPV1, BPV2, and BPV5 are unique in their ability to infect both epithelial and connective tissues. While BPV infections had been documented globally, there was no disease information reported from Taiwan. To investigate whether BPVs are associated with the development of cutaneous papillomas in dairy cattle in Taiwan, in the present study, eight cutaneous papilloma samples from six dairy farms were collected and analyzed by using histopathology, immunohistochemical (IHC) staining, and molecular biology methods. BPV1 and BPV2 were identified, along with a novel BPV sharing 80.9% sequence identity with BPV38. This novel BPV, classified under *Xipapillomavirus*, was detected in both epithelial and mesenchymal cells through in situ hybridization (ISH), suggesting a broader tissue tropism than typical *Xipapillomavirus* infections. These findings provide new insights into BPV diversity and pathogenesis.

## 1. Introduction

Papillomavirus (PV) is an epitheliotrophic pathogen that causes hyperplastic and neoplastic lesions in skin and mucosa [1, 2]. PVs are widely distributed among vertebrates, infecting a broad spectrum of species in mammal, avian, reptile, and Osteichthyes. However, most PV types exhibit strict host-specificity, indicating that cross-species transmission is rare [3]. Although the PV-associated lesions are usually benign and may spontaneously regress along with the elevation of the host's immunity [4, 5], PVs can also lead to malignancies [6–9]. Cervical cancer [6], squamous cell carcinoma (SCC) [7], tricoblastoma [7], Bowenoid in situ carcinoma (BISC), and transitional cell carcinoma (TCC) [8, 9] are linked with specific types of PVs in human, canine, feline, and bovine. Each PV harbors a circular, double-stranded genomic DNA sized approximately 5.7–8.6 kilobase pair (kbp), which encodes seven to nine open reading frames (ORFs) and a noncoding long control region (LCR) [4]. These ORFs encode two capsid proteins (L1 and L2) and four to seven nonstructural proteins (E1, E2, E4, E5, E6, E7, and E8) [4].

According to the definitions provided by the International Committee on Taxonomy of Viruses (ICTV) of PVs, with the sequence identity of L1 ORF <45% could be considered as a different subfamily; <60% a different genus; <90% a different genotype; <98% a different subtype; 98%–99% a different variant [10, 11]. To date, there are 44 genotypes of BPV mainly classified into five genera: *Delta*-, *Xi*-, *Epsilon*-, *Dyoxi*-, and *Dyokappa*-*PV* [12]. While most PVs exhibit strict host specificity and limited tissue tropism [3], BPV1, 2, 5, and 13 have broader host ranges, which have been identified in bovine, horse, giraffe, and donkey [13, 14]. BPV is unique not only in the host range but also in tissue tropism. BPV1, 2, and 5 can infect and transform subepithelial mesenchymal cells, and the subsequent proliferation of both epithelial and subcutaneous connective tissues ultimately leads to the formation of a fibropapilloma [14]. Most BPVs cause benign hyperplastic lesions, however, BPV1 and BPV2 are linked to malignant urinary bladder cancers in cattle [8, 9] and invasive sarcoids in equine [13], while BPV2, 4, 13, and 44 have been associated with alimentary SCCs [15, 16].

It is evidenced that certain BPVs could present not only in the exfoliated keratins but also in body fluids [17]. BPV-induced lesions typically appear on the hairy skin, teats, and urogenital mucosa [14]. Lesions surround oral cavity may cause dysphagia and anorexia, while those on the teats and urogenital mucosa can reduce milk yield, increase mastitis risk, and impair reproduction [18]. Consequently, BPV infections may result in economic losses at the farm level. Moreover, although the PVs are generally host-restricted, specific BPV types with a broad host range could be an invisible threat to both farm animals and wildlife.

Given the importance of BPV in both domestic and wild animals, BPV infections had been reported across several Asian countries [19–21], but relevant reports from Taiwan are limited. In this study, skin lesions clinically diagnosed as papillomas in lactating cattle were collected for BPV detection. Histopathology and polymerase chain reaction (PCR) using two degenerate primer sets were performed, followed by full-genome sequencing and phylogenetic analysis. Immunohistochemical (IHC) and in situ hybridization (ISH) staining further confirmed the presence of BPV in tissue sections.

## 2. Material and Methods

### 2.1. Sample Collection

Eight samples (Case nos. 23-00 to 23-07) were collected from six dairy farms in Taiwan, using excisional (7/8) or scraping (1/8) biopsy. All samples were protruding growths collected from adult dairy cattle. All the masses collected were located on the hairy skin of the flank and were clinically diagnosed as papillomas by veterinarians. They varied in size (diameter of the masses ranged 1–5 cm), but shared a consistent papillomatous appearance. Each tissue sample was divided in half, one fixed in neutral formalin for the histopathological examination while the other was stored at −20°C for further DNA extraction and molecular analysis. However, the sample from Case no. 23-04, collected via scraping biopsy, was submitted only for molecular detection. All procedures involving sample collection from the cattle were performed in accordance with the guidelines of the Institutional Animal Care and Use Committee of National Chung Hsing University.

### 2.2. DNA Extraction and Sequencing Analysis

The nucleic acids were extracted from the fresh tissues by using DNeasy Blood and Tissue Kit (Qiagen, Hilden, Germany). To detect the existence of BPV, two consensus degenerative primer pairs for PVs were used: MY09/11 (forward: 5′-GCMCAGGGWCATAAYAATGG-3′ and reverse: 5′-CGTCCMARRGGAWACTGATC-3′) [22] and CP4/5 (forward: 5′-ATGGTACARTGGGCATWTGA-3′ and reverse: 5′-GAGGYTGCAACCAAAAMTGRCT-3′) [23]. For each PCR reaction, 2 µL of DNA template, 10 µL of amaR OnePCR Buffer (GeneDireX, Taiwan), 0.1–0.2 µM of each primer, and PCR-grade water were mixed to the final volume of 20 µL. The PCR condition was listed as follows: 94°C for 3 min, 35 cycles of 94°C for 30 s, 45°C (MY09/11) or 50°C (CP4/5) for 1 min, 72°C for 45 s, and a 5-min final extension. Electrophoresis was conducted with 1.5% (*w*/*v*) agarose gel and the gel was subsequently stained with SYBR Safe DNA Gel Stain (Invitrogen, Waltham, MA, USA). The expected size of the amplicon of each primer pair was approximately 450 base pair (bp). All the amplicons on expected size were sent to Tri-I Biotech, Inc (Taipei, Taiwan) for Sanger sequencing bidirectionally to confirm the existence of PVs. BigDye Terminator v3.1 Cycle Sequencing Kit (Applied Biosystems, USA) was used for Sanger sequencing and the signals were detected by Applied Biosystems 3730xl DNA Analyzer (Applied Biosystems).

### 2.3. PCR for Full Genome Assembly of BPV1 and BPV2

Six out of eight (6/8) tissue samples tested positive for BPV1 and one (1/8) for BPV2, using conventional PCR technique as described above. For full genome genetic analysis of identified BPV1 and BPV2 isolates, three primer pairs (Table A1) were, respectively, designed to cover the full genome of BPV1 and BPV2 with overlapping regions. The Platinum Green Hot Start PCR Master Mix (Invitrogen) was utilized for long sequence amplification. Each reaction contained 25 µL of Master Mix, 0.2 µM of each primer, 2 µL of DNA template, and PCR-grade water for a total volume of 50 µL. The PCR condition included 94°C for 2 min, 35 cycles of 94°C for 30 s, 50°C for 30 s, and 72°C for 3 min, followed by a final extension of 5 min. The complete genomic assemblies of each case were uploaded to the NCBI database.

### 2.4. De Novo Next-Generation Sequencing (NGS)

One sample was confirmed with PV infection by conventional PCR technique with two degenerative primer sets. However, the preliminary results showed the PV detected in this case had a low sequence identity to the known PVs. Therefore, de novo NGS was used to characterize the full genome of the novel BPV. The DNA extracted from fresh tissue was sent to Tri-I Biotech, Inc (Taipei, Taiwan) for de novo NGS. The DNA sample was fragmented using the enzymatic fragmentation DNA-Seq kit (Takara Bio, CA, USA) and sequenced with the Illumina MiSeq System (Illumina, CA, USA). The quality of raw reads was assessed using NanoPlot v1.28.1. Genome assembly was performed with SPAdes v3.14. The complete genome assembly of this novel BPV was also uploaded to the NCBI database.

### 2.5. IHC Staining

To assess the presence of viral antigen in the lesions, IHC staining was performed. Serial deparaffinization was performed by using the Non-Xylene solution (Muto Chemical, Japan), followed by a rehydration process with graded ethanol from 99% to 80%. Slides were boiled in Tris–EDTA buffer (10 mM Tris, 1 mM EDTA, 0.05% Tween 20, pH 9.0) for 20 min in a steamer to achieve complete antigen-retrieval. After three washes in TBST buffer, the slides were covered with Power Block solution (BioGenex, CA, USA) for 10 min to avoid nonspecific signals. Following three washes in TBST buffer, the mouse anti-HPV antibody (K1H8, ab245950; Abcam, Cambridge, UK) was 1:200 diluted in PBS and subsequently applied onto the slides for 1-h incubation under room temperature. The endogenous peroxidase activity was blocked by incubating the slides with UltraVision Hydrogen Peroxide Block (Thermo Scientific, MA, USA) for 10 min under room temperature. Following three washes in TBST buffer, the slides were covered with the goat anti-mouse IgG antibody conjugated with horseradish peroxidase (HRP; Dako, Agilent Technologies, CA, USA) for 1-h incubation under room temperature. The signals were colorized by using AEC + high sensitivity substrate chromogen (Dako) for 10 min under room temperature. The subtract was removed and the slides were washed in TBST buffer. The counterstaining was performed by immersing the slides into hematoxylin (Muto Chemical) for 1 min and then, washing the slides with running tap water. The signals were visible under microscopic examination.

### 2.6. ISH Staining

ISH staining was performed to better depict the novel BPV in the lesion. The ISH procedure was performed as described by Wang et al. [24], with some modifications. The amplicons produced from PCR with CP4/5 was used to generated the digoxigenin-labeled DNA probe. Subsequently, the embedded tissues were sectioned to obtain 4 µm slides. The slides were deparaffinated and rehydrated as described above in the IHC staining. Antigen retrieval was achieved by soaking the slides in 0.1 N HCl solution followed by enzymic digestion with proteinase K (Qiagen) at 37°C for 15 min. Digoxigenin-labeled probes was added to the slides, then, the slides were heated on the 95°C plate for 6 min, followed by a 16–18 h incubation at 42°C. The endogenous peroxidase activity in the tissue was halted by incubating the slides in 3% H_2_O_2_ for 6 min. The potential nonspecific bindings were blocked by 10% normal goat serum. Probes were detected using the mouse anti-digoxigenin antibody (catalog no.11333062910, Roche) and anti-mouse IgG HRP secondary antibody (Dako). Liquid DAB + substrate chromogen system (Dako) was used to visualize the signals. The tissues on the slides were counterstained with hematoxylin for a better depiction.

### 2.7. Phylogenetic Analysis

Following the guidelines proposed by the ICTV of PV, the L1 ORF, which encodes the major viral capsid protein, serves as the basis for classifying and genotyping PVs. The full-length sequences of the L1 ORF were input to perform the phylogenetic analysis. The genetic information of 44 different genotypes of BPV classified in five genera was included and aligned by MEGA11 software. The phylogenetic tree was generated accordingly using the maximum likelihood method based on the Tamura-Nei model. The sequence identity was calculated using DNAStar software.

## 3. Results

### 3.1. Histological Findings

**Figure 1:**
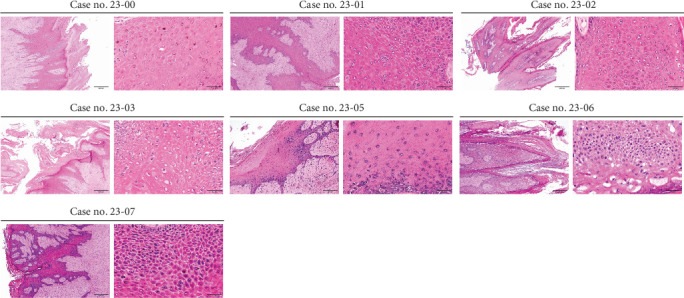
Histopathological findings from seven skin papillomas. The tissue slides were stained with hematoxylin and eosin (H&E). Fibropapillomas (Case nos. 23-00, -01, -03, -05, and -07) and papillomas (Case nos. 23-02 and -06) were diagnosed. Hyperkeratosis was noted in all the cases. Intranuclear inclusions were noticed in Case no. 23-00. Case no. 23-04 was submitted only for molecular detection thus being excluded.

Eight clinical samples diagnosed as skin papillomas by local veterinarians were collected from six dairy farms through excisional or scraping biopsy (Table 1). Histological examination revealed that five samples (Case nos. 23-00, -01, -03, -05, and -07) were fibropapillomas, characterized by notable expansion of the stratum spinosum, connective tissue proliferation, and rete ridge formation (Figure 1). The Case nos. 23-02 and -06 were histologically diagnosed as papilloma (Figure 1). Obvious hyperkeratosis was also noted in all the cases. Eosinophilic intranuclear inclusions with chromatin margination were observed only in Case no. 23-00.

### 3.2. Molecular Diagnosis of PVs by Degenerative Primers

**Figure 2:**
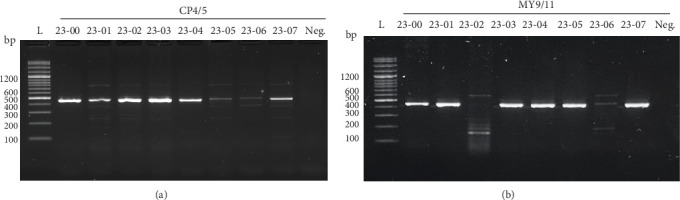
Molecular diagnosis of PVs by two degenerative primers. The viral genome in each sample was amplified by CP4/5 (A) and MY9/11 (B). All the samples were positive for CP4/5, while only seven samples, except Case no. 23-02, were positive for MY9/11. L, ladder; Neg., negative control.

To detect PVs in the sample, two sets of degenerative primers were utilized to amplify the viral genome by PCR technique. All the cases (8/8) exhibited positive bands for CP4/5 (Figure 2A), while seven cases (7/8), excluding Case no. 23-02, showed positive bands for MY9/11 (Figure 2B). Based on the preliminary BLAST analysis on the NCBI database, the PVs in Case nos. 23-00, -01, -03, -04, -05, and -07 were temporarily determined as BPV1, while Case no. 23-06 was classified as BPV2. Interestingly, Case no. 23-02 displayed significant divergence (>10%) from currently known PVs and was temporarily categorized as an “unclassified” genotype.

### 3.3. IHC Staining and ISH Staining

**Figure 3:**
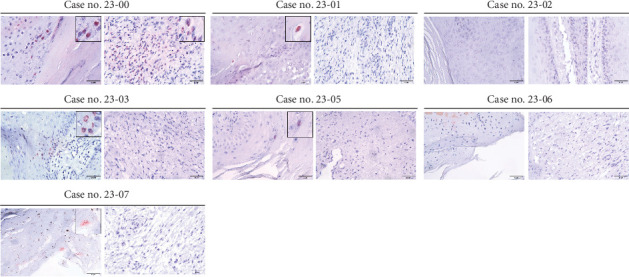
Detection of PVs by IHC staining. The BPV was detected by the anti-HPV antibody and the signals were visualized by the AEC chromogen system. Intranuclear red signals were considered true positive. In Case no. 23-00, the signals were strong in both keratinized epithelial cells and the underlying fibrous connective tissues. In Case nos. 23-01, -03, -05, and -07, only a few positive signals were detected on epithelial cells. In Case nos. 23-02 and -06, there was no signal.

**Figure 4:**
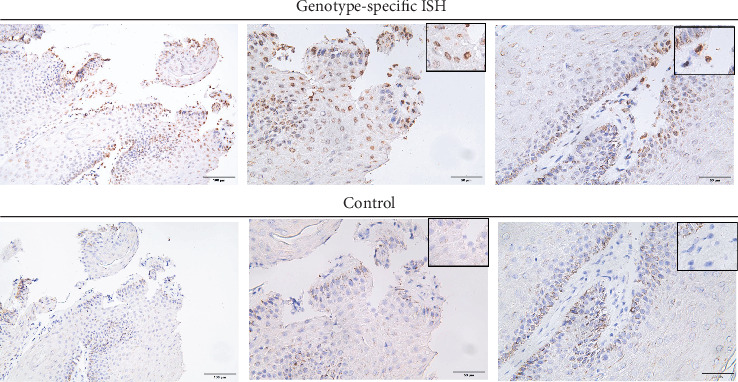
Detection of novel BPV by ISH staining. To assess the presence of the viral genome in the lesion, ISH staining was performed. The positive signals were visualized by the DAB chromogen system and intranuclear brownish signals were considered true positives. Signals were detected mainly in the epithelium, whereas a small proportion of mesenchymal cells also had intranuclear positive signals. A negative control (Control) for excluding the melanin pigments and nonspecific signals was included.

To assess the presence of PV antigens in lesions, IHC staining with anti-HPV antibody (K1H8) was conducted. As shown in Figure 3, the positive signals in Case no. 23-00 appeared in both keratinized epithelial cells and the underlying hyperplastic fibroblasts, with a higher quantity and intensity of signals compared to other cases. Case nos. 23-01, -03, -05, and -07 showed few positive signals in epithelial cells, but none in the connective tissue. Case nos. 23-02 and 23-06 displayed no detectable signals. Since the PV detected in Case no. 23-02 was distant from the known genotype, to further elucidate the causal relationship between the novel BPV and lesion in Case no. 23-02, ISH staining was performed. As shown in Figure 4, the presence of genomic material of BPV in the lesion was clearly depicted by ISH staining. The positive signals mainly appeared at the nucleus of epithelial cells, whereas a small proportion of mesenchymal cells also had intranuclear positive signals.

### 3.4. Phylogenetic Analysis of BPVs

**Figure 5:**
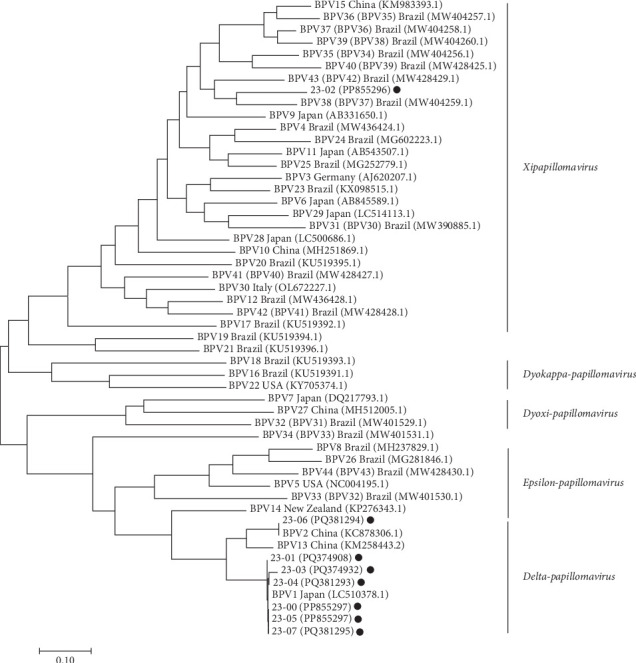
Phylogenetic analysis of BPVs based on the L1 ORF. The full length of L1 ORF of 44 BPV genotypes was aligned using MEGA11 and a phylogenetic tree was generated using the maximum likelihood method based on the Tamura-Nei model. The accession numbers of each strain were labeled. According to the official updated information (https://pave.niaid.nih.gov/index), BPV30 appeared twice in the literature; thus, one was officially renamed BPV31, and the subsequent order was rearranged accordingly. Parentheses indicate the previous names for clarity. The BPVs identified in this study are marked with solid circles and the scale of branch length indicates genetic divergence.

**Figure 6:**
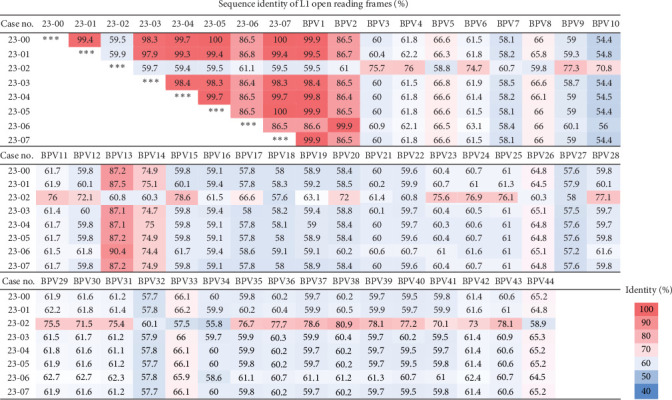
Percentage of sequence identity of BPVs based on L1 ORF. The full length of L1 ORF of 44 BPV genotypes was aligned using MEGA11. All strains analyzed were identical to those used in the phylogenetic study. Sequence identity was calculated using DNAStar and the axial color gradient reflected the percentage of sequence identity between strains.

The full genome assembly of BPV in each case was obtained by overlapping Sanger sequencing with genotype-specific primers (Table A1), while the full genome assembly of the “unclassified” BPV was obtained by performing de novo NGS. The genetic information was uploaded onto the GenBank and the accession numbers for each case were assigned (Table 1). The full-length L1 ORF was aligned with those of reference strains representing 44 distinct BPV genotypes for the phylogenetic analysis. As shown in Figures 5 and 6, Case nos. 23-00, -01, -03, -04, -05, and -07 displayed high sequence identity (>98%) with the BPV1 reference strain (LC510378.1), thus, being classified as the variants of BPV1. Nevertheless, Case no. 23-03 still displayed notable genetic variability (~1.6%) compared to the reference and local strains, suggesting the possibility of local evolution of BPV in herds. Additionally, a BPV2 variant with 99.9% sequence identity to the reference strain (KC878306.1) was also detected. Remarkably, the BPV from Case no. 23-02 showed low sequence identities (55.8–80.9%) with known genotypes and formed a distinct subbranch under the genus of *Xipapillomavirus* (Figure 5), thus, temporarily designated as an “unclassified” genotype. The most closely related strain was BPV38 (MW404259.1), with only 80.9% similarity in the L1 ORF. Following taxonomic rules [10], the unclassified BPV was proposed as a novel genotype (putative BPV genotype 45) within the genus *of Xipapillomavirus*.

### 3.5. Full Genome and Putative Protein Analysis of BPV1 and BPV2

The full genome of BPV1 and BPV2 from the clinical samples was sequenced and assembled. The putative amino acids of each ORF were analyzed and aligned with the reference strains. Although the L1 ORF sequence identities among variants exceeded 98%, several nonsynonymous mutations were still noticed (Table 2). Notably, the BPV1 detected in Case no. 23-03 had the highest variation compared to both the reference strain and other local variants. Case no. 23-07 had a 10-amino acid mutation at the N-terminus of L2. This might indicate that within the same geographic region, BPVs of the same genotypes might independently undergo adaptation and accumulate mutations within their respective cattle populations. Our findings also revealed that the E7 protein of BPV1 exhibited greater conservation, whereas the L2 protein displayed a higher degree of divergence. Notably, all six variants displayed three unique substitutions compared to the reference BPV1 strain: K493R in E1, Q22P in E4, and S452N in L2, indicating potential local evolution of BPV1 in Taiwan. For BPV2, only three amino acid substitutions were identified throughout all viral proteins, including two in E6 and one in L2.

### 3.6. Full Genome and Putative Protein Analysis of the Novel BPV

The full genome of the novel “unclassified” BPV identified in this study was obtained through de novo NGS. As listed in Table 3, the genome spanned 7298 bp and comprised seven putative ORFs: E8, E7, E1, E2, E4, L2, and L1, in referring to BPV38. Amino acid sequence comparisons revealed that the L1, L2, E1, E2, E7, and E8 ORFs of this novel BPV shared 87%, 80%, 82%, 69%, 70.7%, and 76% identity, respectively, with those of BPV38. Several conserved functional motifs of PVs present in BPV38 were also identified in the novel BPV. In the E7 ORF, the retinoblastoma tumor suppressor protein (pRb)-binding motif (L_x_C_x_E) was characterized as L_N_C_E_E, alongside a zinc-binding domain (C_xx_C_x29_C_xx_C) represented as C_YV_C_x29_C_AS_C. Both motifs were recognized regulators of the host's cell cycle. The E8 ORF of the novel BPV contained a hydrophobic transmembrane domain with conserved C-terminal residues (L_x_GWD), which had been linked to immune evasion. Additionally, a conserved ATP-binding site (G_x4_GKS) and an RXL cyclin-binding motif (KRRLL) were both identified in the E1 ORF. The L1 ORF featured two E1-binding sites (E1BS and AACAAT) and one modified E1BS (TAACAA). In the LCR, one modified E1BS (TAACAA) and a canonical E2-binding site (E2BS and ACCG_x4_CGGT) were also found. The ATP-binding site, E1BS, E2BS, and RXL motif were associated with viral genome replication. Notably, no leucine-zipper domain (L_x6_L_x6_L_x6_L_X6_L) was detected in the E1 and E2 ORFs.

## 4. Discussion

Bovine PV (BPV) has been strongly linked to benign and malignant proliferative cutaneous lesions in cattle [1, 2]. Unlike most PVs, which are strictly host-restricted and highly epitheliotrophic, BPVs are unique for interspecies transmission and broad tissue tropism [18, 25]. To investigate the BPV in lactating farms in Taiwan, eight cutaneous papilloma samples from six farms were collected and analyzed through histopathology, molecular diagnosis, and IHC staining as summarized in Table 4. The findings indicated that BPV1 was the most prevalent genotype causing bovine papillomas in Taiwan, although BPV2 was also detected. Notably, a novel BPV genotype (putative BPV45), sharing only 80.9% L1 identity with BPV38, was identified and classified within *Xipapillomavirus* based on full-genome sequencing and phylogenetic analysis. ISH further confirmed its involvement in the development of hyperplastic cutaneous lesions. These findings contribute valuable insights into BPV infections in Taiwan and expand the global understanding of BPVs.

According to published reports, infections with *Delta*-*PV*, particularly BPV1 and BPV2, often involved both epithelial cells and stromal cells, leading to fibropapilloma in cattle or sarcoid in horses [26, 27]. BPV5, an *Epsilon*-*PV*, could also cause mesenchymal transformation [28]. In contrast, *Xipapillomavirus* infections in bovine were typically restricted to epithelial tissues [27]. In this study, a novel BPV in the *Xipapillomavirus* was identified. Despite the lesion caused by this novel BPV was histologically diagnosed as papilloma, the genetic material of this novel BPV was detected in both proliferating epithelial cells and the subepithelial mesenchymal cells by ISH staining, suggesting an expanded tissue tropism for this novel BPV.

BPVs classified within the *Xi*- genus were known for lacking the E6 ORF [27]. Seven putative ORFs and a noncoding LCR were annotated for the novel BPV. The presence of the pRb-binding motif on the E7 suggested its potential to transform epithelial cells [27, 29]. Interestingly, according to the published reports, the absence of the pRb-binding motif in BPV1, 2, and 5 resulted in the lesion manifesting as a fibropapilloma rather than a papilloma [30]. Consistent with this, all BPV1-positive cases in this study were diagnosed as fibropapillomas, while the BPV2-positive case was diagnosed as a papilloma due to minimal stromal proliferation. Although the novel BPV was detected in mesenchymal cells by ISH staining, no significant stromal proliferation was observed, possibly reflecting the early disease stage and supporting previous findings linking the functional pRb-binding motif with reduced fibropapilloma incidence [30]. Additionally, a transmembrane domain and the conserved hydrophilic tail (L_x_GWD) were identified in E8, potentially contributing to immune evasion by modulating MHC expression [31, 32].

The prevalent BPV genotypes vary across countries and even among cattle breeds. Large-scale surveys had shown BPV1 or BPV2 to be predominant in the dairy sector in Egypt, Italy, Turkey, Iraq, Brazil, China, and Japan [19, 21, 33–37], while BPV8 and BPV10 were most common in Germany [38] and BPV7 and BPV8 dominated in Poland [39]. These regional differences in genotype distribution demonstrated the need for local epidemiological investigations. Our study contributed to filling this gap by providing the first molecular and pathological evidence of BPVs in Taiwan. Additionally, many recent reports have highlighted coinfections with different BPV genotypes in lesions [12, 18]. In the present study, BPV1 and BPV2 were detected in different cases by conventional PCR technique with two consensus degenerative primer sets for PVs. By performing conventional PCR with these primers, the possibility of coinfections cannot be completely excluded. Nevertheless, previous research has shown that BPV1 and BPV2 can independently cause hyperplastic or neoplastic lesions in cattle [25, 40, 41], which was consistent with our findings. In Case no. 23-02, the novel BPV was initially detected using degenerative primers, subsequently, de novo NGS was employed to assemble the complete genome and also exclude the possibility of coinfection, further confirming that this novel BPV alone could induce epithelial proliferation leading to papilloma.

To further confirm the presence of BPV in the lesions, IHC staining was performed with the anti-HPV monoclonal antibody (K1H8). Although this antibody exhibited some cross-reactivity [42], positive signals were detected only in the BPV1-positive cases, with no signals observed in BPV2 and the novel BPV cases. Among the IHC-positive tissues, only Case no. 23-00 presented strong positive signals in both epithelial cells and mesenchymal cells, while the other BPV1-positive cases exhibited only a limited number of positive signals. The target of this anti-HPV antibody is the viral capsid protein encoded by the late transcription gene [42]. In early infection stages, viral capsid proteins may not be produced or accumulated in significant quantities, making the detection challenging. Given the lack of a specific antibody, ISH staining with genotype-specific probes was a useful technique to confirm the presence of the BPV in the lesions, highlighting the complementary role of ISH in diagnosing BPV infections.

In summary, a novel BPV genotype, which was suggested to be BPV genotype 45, was identified and characterized from a papilloma in a lactating Holstein. While BPV1 was also detected from hyperplastic lesions from different individuals in the same farm, coinfection of BPVs in the same lesion with the novel BPV was excluded, confirming the independent transforming potential of this novel BPV. Furthermore, IHC and ISH staining revealed that both BPV1 and the novel BPV exhibited tropism for epithelial and connective tissues, expanding current knowledge on BPV tissue specificity. This study also highlighted the circulation of BPV1, BPV2, and the novel BPV in Taiwan, with BPV1 being the most prevalent in skin papillomas.

## Figures and Tables

**Table 1:** Histological findings and information of each case.

Case number	Origin	Histological diagnosis	BPV genotype	GenBank accession no.	L1 ORF gene identity
23-00	Farm A	Fibropapilloma with intranuclear inclusions	BPV1	PP855297	99.9% with BPV1 (LC510378.1)

23-01	Farm B	Fibropapilloma	BPV1	PQ374908	99.5% with BPV1 (LC510378.1)

23-02	Farm A	Papilloma	Unclassified^a^	PP855296	80.9% with BPV38 (MW404259.1)

23-03	Farm C	Fibropapilloma	BPV1	PQ374932	98.4% with BPV1 (LC510378.1)

23-04	Farm D	Not applicable^b^	BPV1	PQ381293	99.8% with BPV1 (LC510378.1)

23-05	Farm E	Fibropapilloma	BPV1	100% identical to PP855297	99.9% with BPV1 (LC510378.1)

23-06	Farm F	Papilloma	BPV2	PQ381294	99.9% with BPV2 (KC878306.1)

23-07	Farm A	Fibropapilloma	BPV1	PQ381295^c^	99.9% with BPV1 (LC510378.1)

^a^According to the taxonomy rules, PVs with L1 ORF sequence identity below 90% were considered different genotypes. The L1 ORF of BPV detected in Case no. 23-02 had only 80.9% to the known BPV, thus, being an unclassified genotype.

^b^Case no. 23-04, collected via scraping, was submitted only for molecular detection.

^c^Although the L1 ORF of Case no. 23-07 was 100% identical to that of Case no. 23-00 and Case no. 23-05, some mutations outside the L1 ORF were observed, resulting in a separate accession number.

**Table 2:** Analysis of amino acid substitutions in BPV1 and BPV2 variants.

BPV1									
Case no.	Total genome length (bp)	Amino acid substitutions comparing to reference BPV1 (LC510378.1)							
		E6	E7	E1	E2	E4	E5	L2	L1
23-00	7946	-	-	K493R N558K	-	Q22P	-	S397N S452N	-

23-01	7945	-	-	K493R	-	Q22P	-	S247P S452N	^a^496-497 KS

23-03	7945	D16E K99Q	-	V54L S109A A117S R137G K493R	S104A I168V S259P T313I N316D	Q22P C40S R101K	L24M T40S G41N	V93A A284G L289I R303K N376H S379P P414L A415P S433T S452N	S31N A55D L176P D270A K351T F441Y

23-04	7947	-	G125A	A2P S272I S281T K493R	D175E K296E V307L N316D	Q22P C40S Q112H	-	K64R R303K P414S S433T S452N	K351T Q398E

23-05	7946	-	-	K493R N558K	-	Q22P	-	S397N S452N	-

23-07	7946	-	-	K493R N558K	-	Q22P	-	^b^VAAGGSPRYTP 65-75 GCCRWITKLHT S397N S452N	-

BPV2									
Case no.	Total genome length (bp)	Amino acid substitutions comparing to reference BPV2 (KC878306.1)							
		E6	E7	E1	E2	E4	E5	L2	L1
23-06	7947	S5T D126H	-	-	-	-	-	D466H	-

*Note:* -, no substitution.

^a^An insertion of two amino acids (─K─S─) at amino acids 496–497 of L1.

^b^A small mutation region (amino acids 65–75) in the L2.

**Table 3:** The analysis of putative amino acid in each ORF of the unclassified novel BPV.

Unclassified BPV								
Case no.	Total genome length (bp)	Potential gene locus (base pairs)						
		E8	E7	E1	E2	E4	L2	L1
23-02	7298	1–129	344–640	633–2441	2383–3600	2903–3334	3614–5206	5217–6731
		**Potential protein length (amino acids)**						
		**E8**	**E7**	**E1**	**E2**	**E4**	**L2**	**L1**
		43	99	603	406	144	531	505

**Table 4:** Overview of diagnostic and analytical methods used for each case.

Case no.	Diagnostic and analytical methods			
	Molecular detection and sequence analysis			Detection of BPVs in tissue
	Conventional PCR with CP4/5	Conventional PCR with MY9/11	Whole-genome sequencing	
23-00	Positive	Positive	Overlapping PCR (BPV1)	IHC
23-01	Positive	Positive	Overlapping PCR (BPV1)	IHC
23-02	Positive	Negative	De novo NGS	ISH
23-03	Positive	Positive	Overlapping PCR (BPV1)	IHC
23-04	Positive	Positive	Overlapping PCR (BPV1)	NE^a^
23-05	Positive	Positive	Overlapping PCR (BPV1)	IHC
23-06	Positive	Positive	Overlapping PCR (BPV2)	NE
23-07	Positive	Positive	Overlapping PCR (BPV1)	IHC

Abbreviations: IHC, immunohistochemical staining; ISH, in situ hybridization; NE, not examined.

^a^Case no. 23-04, collected via scraping, was submitted only for molecular detection.

**Table 5:** The primer pairs used in full genome assembly for BPV1 and BPV2.

Name	Sequence	Length of amplicon (bp)	Cover region on the genome of BPV (bp)
BPV1 seq 1	Forward: 5′GCAAGAACCAATCCATTCTC3′ Reverse: 5′CTGTAGACAGTGTACCAGTT3′	2903	109–3011

BPV1 seq 2	Forward: 5′AGAGTACCACACTCTGTAGT3′ Reverse: 5′GCCTAGCAACAGAATCTGTT3′	3297	2779–6075

BPV1 seq 3	Forward: 5′CTTGGAGGTACTGTAACTGG3′ Reverse: 5′GGTACATGCACCATATCTAC3′	2257	5932–7945 and 1–243 (circular)

BPV2 seq 1	Forward: 5′AGAGGCAATCCTTTCTCAGG3′ Reverse: 5′CCTGTTGTACTAAATCTGGC3′	3050	956–4005

BPV2 seq 2	Forward: 5′CAAACTGTACATGCGCACAG3′ Reverse: 5′TTGCTTCCTGTCATCTGTTG3′	3021	3859–6879

BPV2 seq 3	Forward: 5′ATAGGACTGTGCACAATCCA3′ Reverse: 5′CCATCTCGGTATACAACATG3′	2312	6701–7947 and 1–1065 (circular)

## Data Availability

The data that support the findings of this study are available from the corresponding author upon reasonable request. All relevant data used in the analysis and to draw the conclusions are either included in the article or can be made available upon request.

## References

[1] Munday J. S., Knight C. G., Luff J. A. (2022). Papillomaviral Skin Diseases of Humans, Dogs, Cats and Horses: A Comparative Review. Part 1: Papillomavirus Biology and Hyperplastic Lesions. *The Veterinary Journal*.

[2] International Committee on Taxonomy of Viruses (ICTV) (2023). Virus Taxonomy: 2023 Release.

[3] Frias-De-Diego A., Jara M., Escobar L. E. (2019). Papillomavirus in Wildlife. *Frontiers in Ecology and Evolution*.

[4] Rector A., Van Ranst M. (2013). Animal Papillomaviruses. *Virology*.

[5] Gil da Costa R. M., Peleteiro M. C., Pires M. A., DiMaio D. (2017). An Update on Canine, Feline and Bovine Papillomaviruses. *Transboundary and Emerging Diseases*.

[6] Schiffman M., Doorbar J., Wentzensen N. (2016). Carcinogenic Human Papillomavirus Infection. *Nature Reviews Disease Primers*.

[7] Munday J. S., Kiupel M. (2010). Papillomavirus-Associated Cutaneous Neoplasia in Mammals. *Veterinary Pathology*.

[8] Carvalho T., Pinto C., Peleteiro M. C. (2006). Urinary Bladder Lesions in Bovine Enzootic Haematuria. *Journal of Comparative Pathology*.

[9] Borzacchiello G., Iovane G., Marcante M. L. (2003). Presence of Bovine Papillomavirus Type 2 DNA and Expression of the Viral Oncoprotein E5 in Naturally Occurring Urinary Bladder Tumours in Cows. *Journal of General Virology*.

[10] de Villiers E.-M., Fauquet C., Broker T. R., Bernard H.-U., zur Hausen H. (2004). Classification of Papillomaviruses. *Virology*.

[11] Van Doorslaer K., Chen Z., Bernard H. U. (2018). ICTV Virus Taxonomy Profile: Papillomaviridae. *Journal of General Virology*.

[12] Sauthier J. T., Daudt C., da Silva F. R. C. (2021). The Genetic Diversity of “Papillomavirome” in Bovine Teat Papilloma Lesions. *Animal Microbiome*.

[13] Ogłuszka M., Starzyński R. R., Pierzchała M., Otrocka-Domagała I., Raś A. (2021). Equine Sarcoids—Causes, Molecular Changes, and Clinicopathologic Features: A Review. *Veterinary Pathology*.

[14] Ugochukwu I. C. I., Aneke C. I., Idoko I. S. (2019). Bovine Papilloma: Aetiology, Pathology, Immunology, Disease Status, Diagnosis, Control, Prevention and Treatment: A Review. *Comparative Clinical Pathology*.

[15] Gilio Gasparotto P. H., dos Santos I., Viera Dantas Filho J. (2024). Characterization of Bovine Papillomavirus Types Detected in Cattle Rumen Tissues From Amazon Region, Brazil. *Animals*.

[16] Tsirimonaki E., O’Neil B. W., Williams R., Campo M. S. (2003). Extensive Papillomatosis of the Bovine Upper Gastrointestinal Tract. *Journal of Comparative Pathology*.

[17] Lindsey C. L., Almeida M. E., Vicari C. F. (2009). Bovine Papillomavirus DNA in Milk, Blood, Urine, Semen, and Spermatozoa of Bovine Papillomavirus-Infected Animals. *Genetics and Molecular Research*.

[18] Bocaneti F., Altamura G., Corteggio A., Velescu E., Roperto F., Borzacchiello G. (2016). Bovine Papillomavirus: New Insights Into an Old Disease. *Transboundary and Emerging Diseases*.

[19] Meng Q., Ning C., Wang L. (2021). Molecular Detection and Genetic Diversity of Bovine Papillomavirus in Dairy Cows in Xinjiang, China. *Journal of Veterinary Science*.

[20] Yamashita-Kawanishi N., Chambers J. K., Uchida K. (2021). Genomic Characterisation of Bovine Papillomavirus Types 1 and 2 Identified in Equine Sarcoids in Japan. *Equine Veterinary Journal*.

[21] Peng H., Wu C., Li J. (2019). Detection and Genomic Characterization of Bovine Papillomavirus Isolated From Chinese Native Cattle. *Transboundary and Emerging Diseases*.

[22] Manos M. M., Ting Y., Wright D. K., Lewis A. J., Broker T. R., Wolinsky S. M. (1989). The Use of Polymerase Chain Reaction Amplification for the Detection of Genital Human Papillomavirus. *Cancer Cells*.

[23] Iftner A., Klug S. J., Garbe C. (2003). The Prevalence of Human Papillomavirus Genotypes in Nonmelanoma Skin Cancers of Nonimmunosuppressed Individuals Identifies High-Risk Genital Types as Possible Risk Factors. *Cancer Research*.

[24] Wang H.-Y., Wu M.-C., Chen H.-W. (2023). Isolation, Full Sequence Analysis, and in Situ Hybridization of Pigeon Paramyxovirus-1 Genotype VI.2.1.1.2.2 from Oriental Turtle Doves (*Streptopelia orientalis*). *Poultry Science*.

[25] Munday J. S. (2014). Bovine and Human Papillomaviruses: A Comparative Review. *Veterinary Pathology*.

[26] Doorbar J. (2005). The Papillomavirus Life Cycle. *Journal of Clinical Virology*.

[27] Campo M. S. (2006). Bovine Papillomavirus: Old System, New Lessons?. *Papillomavirus Research: From Natural History to Vaccines and Beyond*.

[28] Borzacchiello G., Roperto F. (2008). *Bovine papillomaviruses*, Papillomas and Cancer in Cattle. *Veterinary Research*.

[29] Kaynarcalidan O., Oğuzoğlu T. Ç. (2021). The Oncogenic Pathways of Papillomaviruses. *Veterinary and Comparative Oncology*.

[30] Narechania A., Terai M., Chen Z., DeSalle R., Burk R. D. (2004). Lack of the Canonical pRB-Binding Domain in the E7 ORF of Artiodactyl Papillomaviruses is Associated With the Development of Fibropapillomas. *Journal of General Virology*.

[31] Ashrafi G. H., Tsirimonaki E., Marchetti B. (2002). Down-Regulation of MHC Class I by Bovine Papillomavirus E5 Oncoproteins. *Oncogene*.

[32] Marchetti B., Ashrafi G. H., Dornan E. S., Araibi E. H., Ellis S. A., Campo M. S. (2006). The E5 Protein of BPV-4 Interacts With the Heavy Chain of MHC Class I and Irreversibly Retains the MHC Complex in the Golgi Apparatus. *Oncogene*.

[33] Hamad M. A., Majeed Al-Shammari A., Odisho S. M., Yaseen N. Y. (2018). Molecular Epidemiology of Bovine Papillomatosis and Identification of Three Genotypes in Central Iraq. *Intervirology*.

[34] Bertagnolli A. C., Bezerra A. V. A., Santos R. N. (2020). Clinicopathological Characteristics and Papillomavirus Types in Cutaneous Warts in Bovine. *Brazilian Journal of Microbiology*.

[35] Ata E. B., Allam A. M., Elbayoumy M. K., Mahmoud M. A. E. (2021). Electron Microscopy and Phylogenetic Analysis of Bovine Papillomavirus Infection in Cattle From Four Egyptian Governorates. *Tropical Animal Health and Production*.

[36] Dagalp S. B., Dogan F., Farzanı T. A., Salar S., Bastan A. (2017). The Genetic Diversity of Bovine Papillomaviruses (BPV) From Different Papillomatosis Cases in Dairy Cows in Turkey. *Archives of Virology*.

[37] Grindatto A., Ferraro G., Varello K. (2015). Molecular and Histological Characterization of Bovine Papillomavirus in North West Italy. *Veterinary Microbiology*.

[38] Schmitt M., Fiedler V., Müller M. (2010). Prevalence of BPV Genotypes in a German Cowshed Determined by a Novel Multiplex BPV Genotyping Assay. *Journal of Virological Methods*.

[39] Pyrek P., Bednarski M., Popiel J., Siedlecka M., Karwańska M. (2023). Genetic Evaluation of Bovine Papillomavirus Types Associated With Teat Papillomatosis in Polish Dairy Cattle With the Report of a New Putative Type. *Pathogens*.

[40] Yuan Z. Q., Gault E. A., Gobeil P., Nixon C., Campo M. S., Nasir L. (2008). Establishment and Characterization of Equine Fibroblast Cell Lines Transformed in Vivo and in Vitro by BPV-1: Model Systems for Equine Sarcoids. *Virology*.

[41] Corteggio A., Altamura G., Roperto F., Borzacchiello G. (2013). Bovine Papillomavirus E5 and E7 Oncoproteins in Naturally Occurring Tumors: Are Two Better Than One?. *Infectious Agents and Cancer*.

[42] Iwasaki T., Sata T., Sugase M. (1992). Detection of Capsid Antigen of Human Papillomavirus (HPV) in Benign Lesions of Female Genital Tract Using Anti-HPV Monoclonal Antibody. *The Journal of Pathology*.

